# Reducing the burden of breast implant infections through a structured multidisciplinary pathway

**DOI:** 10.1016/j.jpra.2026.05.038

**Published:** 2026-05-28

**Authors:** Roberta Comunian, Stefano Vaccari, Edoardo Caimi, Riccardo Di Giuli, Flavio Bucci, Mattia Federico Cavallero, Simone Furlan, Alessandra Veronesi, Valeria Bandi, Barbara Catania, Marco Klinger, Maddalena Casana, Diletta Barbanotti, Davide Bavaro, Francesco Klinger, Michele Bartoletti, Valeriano Vinci

**Affiliations:** a Department of Medical Biotechnology and Translational Medicine BIOMETRA, Reconstructive and Aesthetic Plastic Surgery School, University of Milan, Milan, Italy; b Humanitas University, Department of Biomedical Sciences, Via Rita Levi Montalcini 4, Rozzano, Milan, Italy; c IRCCS Humanitas Research Hospital, Via Manzoni 56 20089 Rozzano, Milan, Italy; d Infectious Disease Unit, IRCCS Humanitas Research Hospital, Via Manzoni 56, Rozzano, 20089 Milan, Italy; e Department of Health Sciences, Ospedale San Paolo, University of Milan, Via Antonio di Rudinì, 8, 20142, Milan, Italy

**Keywords:** Breast cancer, Breast reconstruction, Breast infection, Multidisciplinary consult, Breast implant infection, Infection incidence, Breast reconstruction complications

## Abstract

**Background:**

Breast implant–related infections are major complications of implant-based breast reconstruction, leading to implant loss, delays in adjuvant oncologic therapies, prolonged hospitalization, and increased healthcare costs. Despite their impact, management strategies remain heterogeneous, and evidence supporting structured preventive approaches is limited. This study evaluated the effect of a structured multidisciplinary pathway (CONMUL) on infection incidence and clinical outcomes.

**Methods:**

A single-center retrospective cohort study included patients who underwent mastectomy followed by implant-based breast reconstruction between January 2022 and December 2025. From January 2024, a multidisciplinary protocol integrating plastic surgeons and infectious disease specialists was progressively implemented, including standardized preventive measures, early specialist consultation, and shared management pathways. The primary outcome was the trend in postoperative infection incidence before and after CONMUL implementation. Secondary outcomes included explantation rates, implant salvage, reinfection, and time to explantation.

**Results:**

Overall, 927 patients (1102 reconstructed breasts) were included, and 98 breasts (8.9%) developed postoperative infection. Annual infection incidence peaked at 11.42% in 2023 and declined following CONMUL implementation, reaching 5.15% in 2025, corresponding to approximately 15 fewer infections per year. Once infection occurred, explantation remained the most frequent outcome, with no significant differences between multidisciplinary and standard management. Time to explantation and reinfection rates were comparable between groups.

**Conclusions:**

A structured multidisciplinary pathway was associated with a clinically meaningful reduction in postoperative breast implant infection incidence. While multidisciplinary management did not significantly change outcomes after infection onset, it functioned as an effective preventive, system-level strategy without compromising clinical safety.

## Introduction

Breast implant–related infections are among the most consequential complications of implant-based breast reconstruction and a major driver of reconstructive failure. Their impact extends beyond the surgical setting: they delay adjuvant oncologic treatments, increase reoperations and hospitalizations, and impair quality of life during an already vulnerable phase of cancer care. When they occur, they may produce severe local and systemic complications, frequently necessitating implant removal and compromising both immediate and delayed reconstructive options.

Reported incidence rates vary widely, from 1% to 35.4%,^1^, ^2^, ^3^, ^4^ reflecting heterogeneity in patient populations, surgical techniques, and perioperative management, and the lack of standardized preventive and therapeutic strategies. Risk is influenced by multiple patient- and treatment-related factors, including chemotherapy,^5^ radiotherapy, BMI,^6^ tobacco use, disruption of the lymphovascular system, use of acellular dermal matrices (ADMs), and prolonged surgical drain placement.^6^^,^^7^ Both direct-to-implant (DTI) and two-stage reconstructions are affected, the latter carrying additional risk from repeated outpatient expansions. Beyond clinical sequelae, these infections generate a substantial economic burden through prolonged hospital stays, repeated procedures, long-term antibiotic therapy, and intensified follow-up.^6^^,^^8^

Clinical presentation is heterogeneous: erythema, edema, wound dehiscence, periprosthetic fluid collections, fever, and pain^3^, and none of these signs are pathognomonic, overlapping with conditions such as Red Breast Syndrome, seroma, breast implant–associated anaplastic large cell lymphoma (BIA-ALCL), and nonspecific postoperative inflammation.^2^^,^^10^ By timing, infections are classified as acute (within 6 weeks of surgery) or late (more than 6 months postoperatively).^3^ Microbiological cultures remain the diagnostic cornerstone for guiding targeted antibiotic therapy, despite turnaround delays.

Preventive strategies are therefore paramount, as reducing incidence is the most effective means of limiting downstream complications, oncologic delays, and economic costs. They rely on strict intraoperative sterility and meticulous device handling. Preoperative antibiotic prophylaxis is well established,^11^ whereas the benefit of routine postoperative prophylaxis remains controversial.^12^^,^^13^

In practice, infections are most often managed initially with empiric broad-spectrum antibiotics prescribed by the treating surgeon.^14^ This non-standardized, experience-driven approach can contribute to treatment failure, prolonged infection, and antimicrobial resistance^15^ More aggressive strategies include implant removal or exchange,^16^^,^^17^ with variable outcomes. Definitive explantation is often the most appropriate option given the frequent association between prosthetic infections and biofilm, which once established must be eradicated to prevent persistent infection and capsular contracture.^18^^,^^19^

Despite the clinical relevance of these infections, therapeutic approaches remain heterogeneous^14^: no universally accepted algorithm exists, official guidelines are lacking, and evidence supporting early, structured multidisciplinary interventions is limited. This absence of standardization contributes to inconsistent outcomes, unnecessary implant loss, delays in oncologic therapy, and escalating healthcare costs.

We hypothesized that a structured multidisciplinary protocol integrating plastic surgeons and infectious disease specialists could reduce the incidence of breast implant infections and optimize their management, thereby decreasing the overall clinical, economic, and patient-centered burden. This study reports the outcomes observed following adoption of such a pathway at our institution.

## Materials and methods

### Study design

This single-center retrospective cohort study was conducted at the Department of Plastic and Reconstructive Surgery, Humanitas Hospital, Milan, Italy, in accordance with the Declaration of Helsinki and ICH-GCP. Consecutive patients who underwent mastectomy followed by implant-based breast reconstruction (IBR) between January 2022 and December 2025 and developed a postoperative infection (early or late) were included. Infection was defined by clinical signs such as erythema, edema, breast pain, fever, wound dehiscence, purulent drainage, and/or periprosthetic fluid collection, supported when available by microbiological cultures, laboratory findings, and imaging. Because microbiological confirmation was not immediately available in all cases, early management was guided by clinical suspicion.

No changes in surgical staff, institutional setting, or patient selection criteria occurred during the study period other than the progressive implementation of the CONMUL pathway

### Patient eligibility

All patients undergoing IBR at our institution during the study period and followed for postoperative infectious outcomes were included; the infected-case subgroup analysis was restricted to patients with a clinically suspected and microbiologically confirmed breast implant infection.

Exclusion criteria were autologous reconstruction, absence of suspected or confirmed infection, and incomplete or unclear clinical records.

### Data extraction

Data were extracted from preoperative, intraoperative, and postoperative medical records. Collected variables included general health status, oncologic treatments (chemotherapy, radiotherapy), reconstructive details (technique, implants vs. tissue expanders, ADM use), clinical presentation (early ≤ 6 weeks vs. late > 6 weeks), microbiological findings when available, and administered antibiotic therapy.

### Multidisciplinary CONMUL pathway for infection assessment and management

**Fig. 1:**
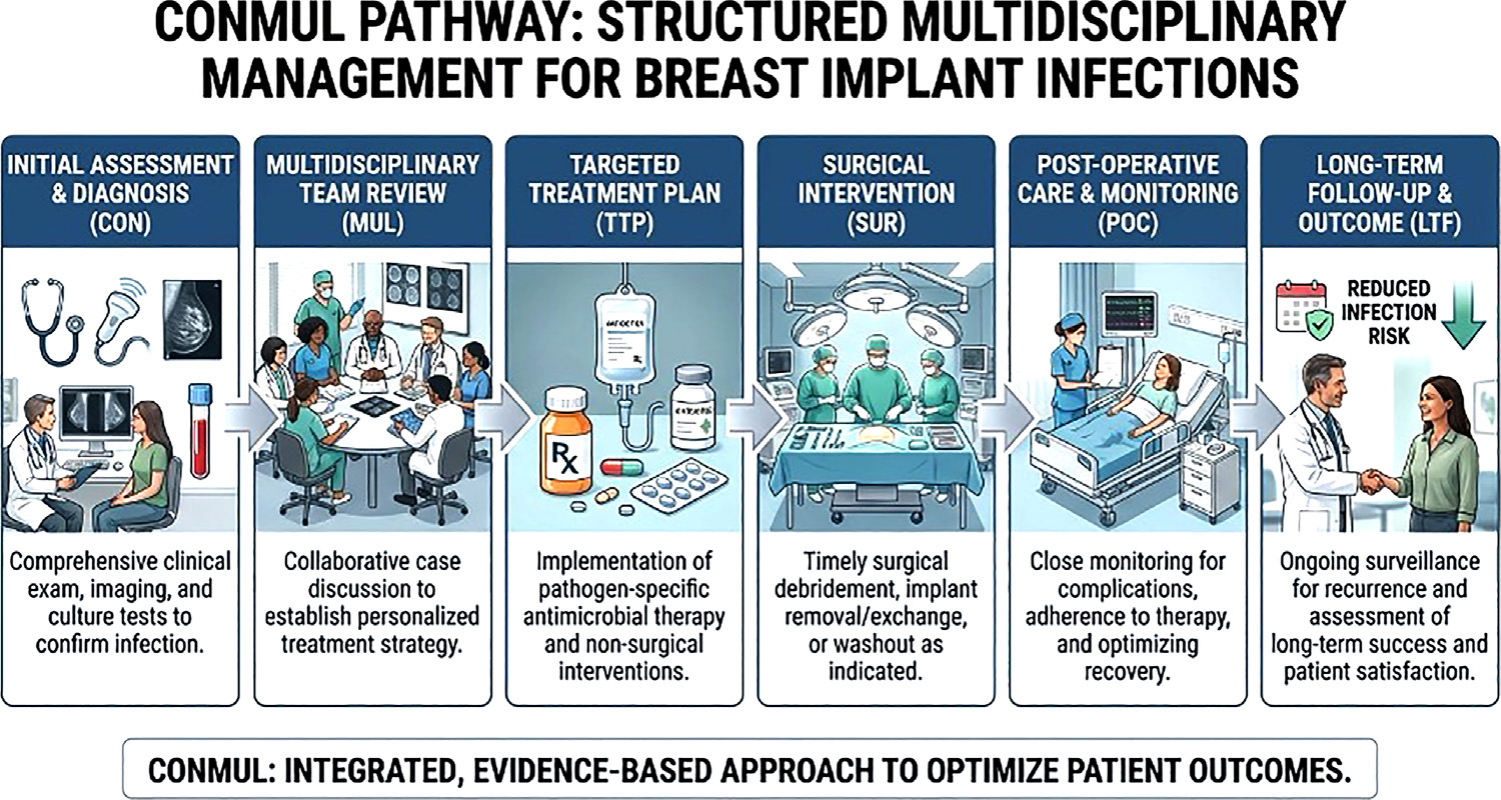
The figure depicts the structured clinical workflow introduced by the CONMUL pathway, including early standardized assessment and diagnosis, multidisciplinary team review, targeted antimicrobial therapy, and timely surgical intervention when indicated. Postoperative monitoring and long-term follow-up are integrated to optimize recovery, detect recurrence, and assess reconstructive outcomes. Overall, CONMUL provides a standardized, evidence-based approach aimed at reducing infection incidence and the associated clinical and healthcare burden.

From January 2024, multidisciplinary consultation with infectious disease specialists was progressively integrated into the management of postoperative breast infections (Fig. 1), supported by internal clinical care pathways (PDTA) for patients undergoing mastectomy and IBR.

Preoperatively, all patients underwent a nasal swab for methicillin-sensitive/methicillin-resistant Staphylococcus aureus (MSSA/MRSA) colonization; carriers followed a decolonization protocol of daily chlorhexidine washes and intranasal mupirocin three times daily, starting five days before surgery. Perioperative antibiotic prophylaxis consisted of intravenous cefazolin 2 g 30 minutes before surgery, or intravenous clindamycin 600 mg in patients with beta-lactam allergy, renewed at 4 hours if surgery was prolonged. The surgical field was prepared with surgical chlorhexidine antisepsis and sterile disposable drapes; before implant positioning the prosthetic pocket was irrigated with 10% povidone-iodine and rinsed with saline, and the implant was immersed in the same solution. After a sterile glove change, the primary surgeon proceeded with device placement.

Patients considered at higher risk of progression based on clinical, laboratory, and radiological findings were discussed jointly by a plastic surgeon and an infectious disease specialist. At the first signs of infection (e.g., fever or erythema), patients were evaluated jointly in the outpatient setting, and the team decided between conservative management (antibiotic therapy with close follow-up) and direct implant removal; when conservative treatment failed, explantation was performed. Patients continued to be reviewed jointly at each subsequent visit, with email communication used for test results and clinical updates.

An emergency-department fast-track pathway ensured that patients with acute infectious manifestations bypassed the general ER and were evaluated immediately by the on-duty infectious disease specialist, followed by plastic surgery consultation. Management of patients who had already undergone explantation was standardized through multidisciplinary discussion to define timing of reimplantation and any adjunctive therapies.

### Outcomes

The primary outcome was the postoperative infection rate trend before and after introduction of the multidisciplinary approach. Secondary outcomes were management outcomes (explantation, salvage, immediate substitution) and revision surgery for recurrent infection, stratified by reconstruction technique and by infectious disease consultation.

### Statistical analysis

Categorical variables are reported as n (%) and continuous variables as mean ± SD or median (IQR) depending on distribution, assessed using the Shapiro-Wilk test. Associations were tested with Fisher's exact test. Analyses were performed in Stata 19 SE (StataCorp, College Station, TX, USA); p < 0.05 was considered significant.

## Results

**Fig. 2:**
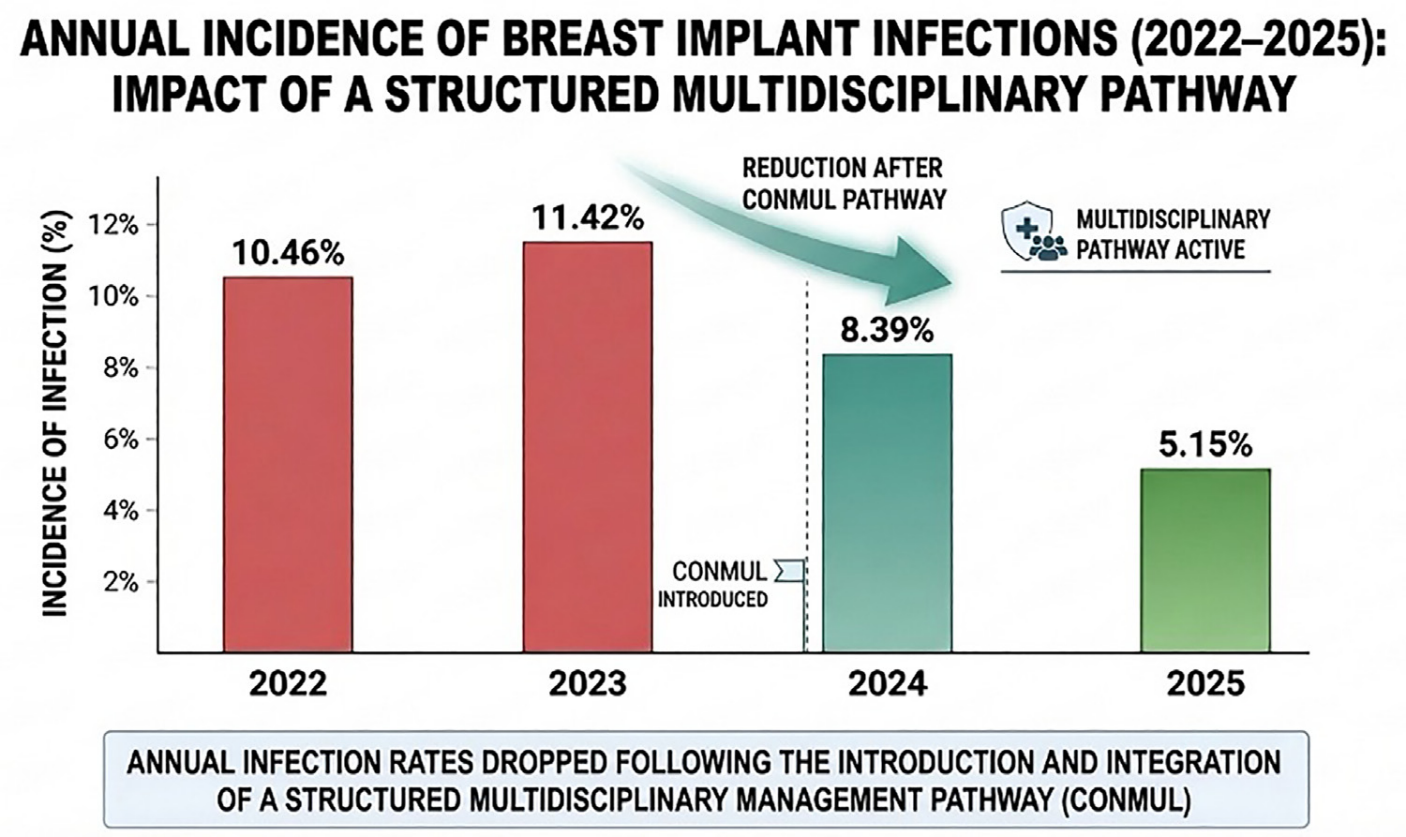
The figure shows a progressive reduction in postoperative breast implant infection rates following the introduction of the CONMUL structured multidisciplinary pathway. After peaking in 2023, infection incidence declined markedly in 2024 and further decreased to 5.15% in 2025, coinciding with full integration of the multidisciplinary protocol. These data highlight the preventive, system-level impact of CONMUL in reducing the overall burden of implant-related infections.

During the study period, 927 patients underwent IBR, for a total of 1102 operated breasts; 98 (8.89%) developed infection and met the inclusion criteria. Within the cohort, 370 patients (44.47%) underwent expander-based reconstruction, 307 (36.90%) unilateral reconstruction, and 155 (18.63%) bilateral reconstruction. Annual infection incidence was 34/325 (10.46%) in 2022, 25/219 (11.42%) in 2023, 25/286 (8.39%) in 2024, and 14/272 (5.15%) in 2025, with a declining trend following implementation of the CONMUL pathway (Fig. 2).

Of the 98 infected patients, 52 (53.1%) were managed with a standard, non-multidisciplinary approach (non-CONMUL group) and 46 (46.9%) within the multidisciplinary pathway (CONMUL group). Overall, implant explantation was required in 84 cases (85.7%) and implant preservation (successful salvage) achieved in 14 (14.3%): 45/52 (86.5%) vs. 39/46 (84.8%) explantations and 7/52 (13.5%) vs. 7/46 (15.2%) salvages in the non-CONMUL and CONMUL groups, respectively. No significant difference in explantation rates was observed (χ² = 0.0615, df = 1, p = 0.804; Fisher's exact p = 1.000); the odds ratio for explantation in the CONMUL vs. non-CONMUL group was 0.87 (95% CI 0.28–2.69) (Table 2).

**Fig. 3:**
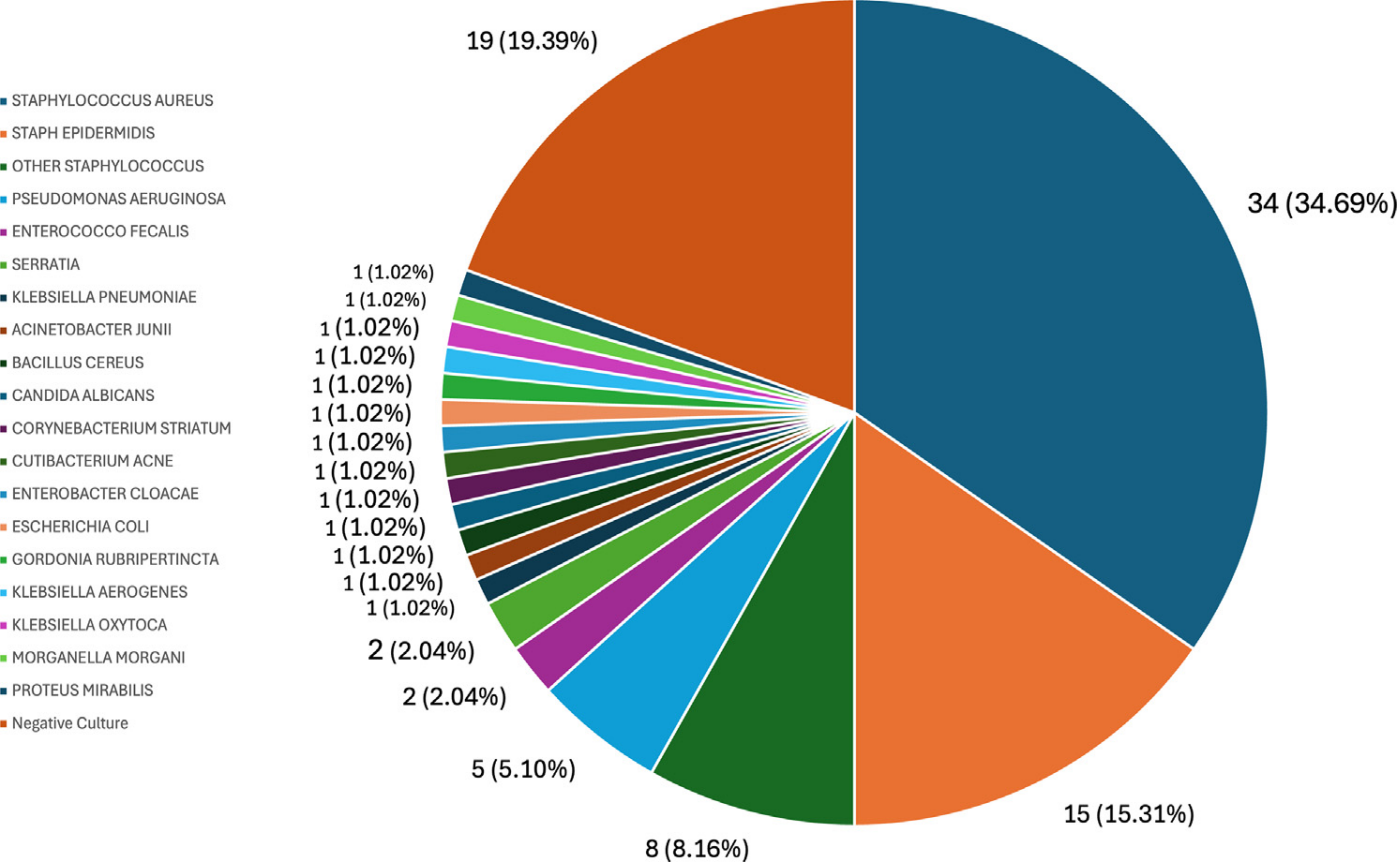
Pie chart illustrating the distribution of pathogens isolated from our infected patient cohort.

Among the 98 infected patients, the most frequently isolated pathogen was *Staphylococcus aureus* (n=34, 34.69%), followed by *Staphylococcus epidermidis* (n=15, 15.31%), other *Staphylococcus* species (n=8, 8.16%), and *Pseudomonas aeruginosa* (n=5, 5.10%); cultures were negative in 19 cases (19.39%). Fig. 3 reports the detailed microbiological spectrum.

Clinical outcomes were categorized as explantation, implant salvage, or immediate implant substitution. Overall, explantation was the most frequent outcome (76/98, 77.6%), with salvage and immediate substitution each in 11 patients (11.2%). In the non-CONMUL vs. CONMUL groups, respectively: explantation 38/52 (73.1%) vs. 38/46 (82.6%), salvage 5 (9.6%) vs. 6 (13.0%), and immediate substitution 9 (17.3%) vs. 2 (4.3%). The distribution did not differ significantly between groups (χ² = 4.19, df = 2, p = 0.123; Fisher's exact p = 0.142).

Reconstruction-type distribution differed significantly between groups (Table 1). Overall, 40 patients (40.8%) underwent direct-to-implant (DTI) reconstruction and 58 (59.2%) a two-stage expander–implant reconstruction (counting only the first-stage expander positioning). DTI was performed in 16/52 (30.8%) of the non-CONMUL group vs. 24/46 (52.2%) of the CONMUL group, with two-stage reconstruction in the remainder (36/52, 69.2% vs. 22/46, 47.8%; χ² = 4.63, df = 1, p = 0.031; Fisher's exact p = 0.040).

**Table 1:** Baseline demographic, clinical, and reconstructive characteristics of patients who developed postoperative infection following implant-based breast reconstruction, stratified by management approach.

Variable	Overall (n = 98)	Non-CONMUL (n = 52)	CONMUL (n = 46)	*p-* value
Age, years (mean ± SD)	53.1 ± 9.5	54.7 ± 8.9	51.2 ± 9.8	0.073
BMI (mean ± SD)	25.2 ± 4.7	24.8 ± 4.2	25.7 ± 5.2	0.339
Smoking status (%)	2 (2.0%)	1 (1.9%)	1 (2.2%)	1.000
Diabetes mellitus (%)	36 (36.7%)	30 (57.7%)	6 (13.0%)	0.309
Chemotherapy (%)	38 (38.8%)	22 (42.3%)	16 (34.8%)	0.867
Radiotherapy (%)	19 (19.4%)	10 (19.2%)	9 (19.6%)	1
ADM use (%)	0 (0%)	0 (0%)	0 (0%)	1
Direct-to-implant reconstruction (%)	40 (40.8%)	16 (30.8%)	24 (52.2%)	**0.052**
Two-stage reconstruction (%)	58 (59.2%)	36 (69.2%)	22 (47.8%)	**0.052**
MSSA/MRSA positive (%)	14 (14.3%)	9 (17.3%)	5 (10.9%)	**0.402**
MSSA/MRSA negative (%)	61 (62.2%)	30 (57.7%)	30 (65.2%)	**0.579**
Nasal swab not performed (%)	23 (23.5%)	13 (25.0%)	11 (23.9%)	**1.000**

**Table 2:** Clinical outcomes following postoperative breast implant infection, stratified by management approach.

Outcome	Overall (n = 98)	Non-CONMUL (n = 52)	CONMUL (n = 46)	*p-*value
Explantation	76 (77.6%)	38 (73.1%)	38 (82.6%)	0.123*
Implant salvage	11 (11.2%)	5 (9.6%)	6 (13.0%)	—
Immediate implant substitution	11 (11.2%)	9 (17.3%)	2 (4.3%)	—
Reinfection	7 (7.1%)	4 (7.7%)	3 (6.5%)	0.822

*p-value refers to comparison of overall outcome distribution between groups (χ² test).

Time to explantation was analyzed in the 85 patients who required implant removal (46 non-CONMUL, 39 CONMUL; Table 3). Both groups showed non-normal distributions on Shapiro–Wilk testing (non-CONMUL: W = 0.828, p < 0.001; CONMUL: W = 0.777, p < 0.001), so both parametric and non-parametric tests were applied. Mean time to explantation was 156 ± 140 days (median 93.5, IQR 175, range 0–556) in the non-CONMUL group and 178 ± 180 days (median 100, IQR 193, range 17–720) in the CONMUL group, with no significant difference (mean difference −21.8 days, 95% CI −91.0 to 47.3; t = −0.628, df = 83, p = 0.532; Mann–Whitney U = 881, p = 0.891).

**Table 3:** Time interval between primary implant-based breast reconstruction and implant explantation in patients requiring implant removal, stratified by management approach

Variable	Non-CONMUL (n = 46)	CONMUL (n = 39)	*p*-value
Mean ± SD (days)	156 ± 140	178 ± 180	0.532
Median (IQR), days	93.5 (175)	100 (193)	0.891
Range, days	0–556	17–720	—

Reinfection occurred in 7/98 patients (7.1%) overall — 4/52 (7.7%) in the non-CONMUL group and 3/46 (6.5%) in the CONMUL group — with no significant difference between groups (χ² = 0.050, df = 1, p = 0.822; Fisher's exact p = 1.000). The odds ratio for reinfection in the CONMUL vs. non-CONMUL group was 0.84 (95% CI 0.18–3.95).

## Discussion

Postoperative infection remains a clinically significant and challenging complication of IBR, contributing to reconstructive failure, delays in adjuvant oncologic therapies, increased healthcare costs, and psychological distress.^16^ Despite advances in surgical and perioperative management, treatment remains heterogeneous and standardized algorithms are lacking.^14^ Multidisciplinary models involving infectious disease specialists have been increasingly promoted, but their impact on clinical outcomes has not been clearly defined.

After infection onset, explantation was the predominant endpoint. No significant differences were observed between the surgeon-driven and CONMUL groups in overall explantation rates or in the distribution of explantation, salvage, and immediate substitution. Once infection is established, the current multidisciplinary approach does not substantially modify the final reconstructive outcome.

This reflects the biological complexity of implant-related infections rather than a limitation of the CONMUL model. Prosthetic breast infections are multifactorial, involving host susceptibility, surgical factors, microbiology, and biofilm on the implant surface.^18^^,^^19^ Once mature biofilm is established, even optimized antibiotic regimens and close multidisciplinary monitoring may be insufficient, making explantation the safest definitive option.^16^^,^^17^

Time to explantation did not differ significantly between groups; CONMUL patients showed a numerically longer interval, but this is clinically reassuring, indicating that multidisciplinary management does not delay necessary surgical decisions nor expose patients to prolonged uncontrolled infection, while allowing careful monitoring and conservative attempts when appropriate.

Reinfection rates were low across the cohort and did not differ between groups. The CONMUL pathway was not associated with increased reinfection risk despite a more structured and, in selected cases, conservative approach, supporting its safety and the role of infectious disease specialists in antibiotic stewardship and follow-up.^9^^,^^15^

A higher proportion of DTI reconstructions was observed in the CONMUL cohort. This likely reflects temporal changes in surgical practice rather than an effect of multidisciplinary management: non-CONMUL patients were predominantly treated in 2022–2023 when two-stage reconstruction was more common, whereas the CONMUL group was treated in 2024–2025, coinciding with a broader institutional shift toward immediate implant-based reconstruction.

The most clinically relevant finding emerges at the population level. Infection incidence peaked at 11.42% in 2023, declined after CONMUL introduction in 2024, and reached 5.15% in 2025. The reduction from 10.46% (2022) to 5.15% (2025) corresponds to approximately 15 fewer infections per year, or nearly 60 over the study period. At a conservative estimate of €4,000–€8,000 per infection avoided,^20^^,^^21^ this represents roughly €60,000–€120,000 saved annually in direct hospital costs alone, excluding indirect savings from reduced readmissions, reoperations, and length of stay.

The reduction became more pronounced over time, with the lowest rate in 2025 when the pathway was fully integrated into routine practice. Although causality cannot be definitively established given the retrospective design, the magnitude, consistency, and temporal association support a preventive effect of the CONMUL model and indicate that multidisciplinary interventions exert their greatest benefit as system-level preventive strategies rather than rescue tools after infection.

The effectiveness of multidisciplinary management should therefore be assessed on its ability to reduce infection incidence and interrupt the cascade of complications, not solely on downstream outcomes such as explantation or salvage rates. From this perspective, CONMUL functions primarily as a preventive, organizational intervention — improving early recognition, standardization of care, antibiotic stewardship, and interdisciplinary communication.

Importantly, no increase in explantation rates, reinfections, or management delays was observed, confirming that multidisciplinary management is not only effective in reducing infection incidence but also safe.

The CONMUL pathway exerts its strongest effect at the preventive level; once infection is established, its impact on reconstructive recovery remains limited. Recent literature points to faster, more aggressive diagnostic strategies. Caputo et al.^22^ described a salvage-oriented protocol combining preoperative screening, optimized perioperative prophylaxis, ultrasound-guided assessment of periprosthetic fluid, and rapid molecular diagnosis alongside standard cultures, with confirmed infections promptly managed by implant removal, pocket debridement, and targeted therapy.^22^ The same group subsequently reported that a multiplex-PCR assay yielded microbiological results within hours, compared with the 4–5 days typically required for conventional cultures, and showed high concordance with standard culture.^23^ A prospective study of electrochemical impedance spectroscopy in periprosthetic fluids further demonstrated detection of viable microorganisms within approximately 9 minutes and full concordance with culture in distinguishing Gram-positive from Gram-negative infections, although without species-level identification.^24^ Taken together, these findings suggest that future refinements of the CONMUL pathway could incorporate earlier microbiological stratification — particularly in patients with periprosthetic fluid collections or features suggestive of treatment failure — to better distinguish those suitable for conservative management from those requiring earlier surgical escalation.^22^, ^23^, ^24^

Beyond clinical outcomes, the CONMUL model offers medico-legal advantages increasingly relevant in contemporary reconstructive practice. Formal, documented involvement of infectious disease specialists transforms infection management from a surgeon-dependent decision into a shared multidisciplinary responsibility, enhancing traceability, transparency, and defensibility of clinical decisions. Anchoring management to collegial discussion, standardized pathways, and evidence-based antibiotic strategies reduces professional isolation and mitigates exposure associated with adverse outcomes. The joint evaluation by two specialists also reinforces informed consent and shared decision-making, improving patient acceptance of potential outcomes including implant loss. While multidisciplinary care is well established in oncology and other high-risk settings,^25^^,^^26^ its structured application to IBR introduces a novel framework strengthening both clinical governance and medico-legal protection.

## Conclusion

In this retrospective cohort study, the introduction of a structured multidisciplinary management pathway for post-reconstruction breast infections was associated with a significant and progressive reduction in the overall incidence of postoperative infections over time. These preliminary findings suggest that the primary value of multidisciplinary collaboration lies in infection prevention and early control rather than in altering downstream reconstructive endpoints. By enabling standardized protocols, early infectious disease involvement, and optimized antibiotic stewardship, the CONMUL model appears to function as an effective system-level intervention that reduces the global burden of postoperative breast infections. Multidisciplinary management should therefore be regarded as a key component of contemporary implant-based breast reconstruction pathways, by fostering collaboration while streamlining surgical responsibilities, enhancing medical-legal safety, and reassuring patients through the involvement of two specialists; this new approach represents a promising future direction for infection management in reconstructive breast surgery, with the potential to redefine standards of care in implant-based breast reconstruction.

## Funding

None.

## Ethical approval

Approved by the local hospital ethical board.

## Financial disclosure

Authors have nothing to disclose.

## Declaration of generative AI use

During the preparation of this work the author(s) used FigureLabs AI in order to generate all of the graphical figures of this manuscript in order to better represent the information within the manuscript. After using this tool/service, the author(s) reviewed and edited the content as needed and take(s) full responsibility for the content of the published article.

## Declaration of competing interest

None declared.

## References

[1] Alderman A.K., Wilkins E.G., Kim H.M., Lowery J.C. (June 2002). Complications in postmastectomy breast reconstruction: two-year results of the Michigan breast reconstruction outcome study. Plast Reconstr Surg.

[2] Washer L.L., Gutowski K. (Mar. 2012). Breast implant infections. Infect Dis Clin North Am.

[3] Lalani T. (Dec. 2018). Breast implant infections. Infect Dis Clin North Am.

[4] Xue A., Kania K., Brown R., Bullocks J., Hollier L., Izaddoost S. (May 2016). Salvage of infected prosthetic breast reconstructions. Semin Plast Surg.

[5] Lee K.-T., Bae J., Jeon B.J., Pyon J.K., Mun G.-H., Bang S.I. (Apr. 2021). Adjuvant chemotherapy in two-stage tissue expander/implant breast reconstruction: does it affect final outcomes?. Ann Surg Oncol.

[6] Vaccari S. (Nov. 2023). Implant-based breast reconstruction: impact of body mass index on postoperative complications and aesthetic results: A 5-year, single-center study. Aesthet Surg J.

[7] Viola G.M. (May 2016). Improving antimicrobial regimens for the treatment of breast tissue expander-related infections. Plast Reconstr Surg - Glob Open.

[8] Frois A.O. (2018). The role of antibiotics in breast pocket irrigation and implant immersion: A systematic review. Plast Reconstr Surg - Glob Open.

[9] Viola G.M. (June 2016). Salvaging the infected breast tissue expander: A standardized multidisciplinary approach. Plast Reconstr Surg - Glob Open.

[10] Azouz V., Mirhaidari S., Wagner D.S. (May 2018). Defining infection in breast reconstruction: A literature review. Ann Plast Surg.

[11] Phillips B.T., Halvorson E.G. (Oct. 2016). Antibiotic prophylaxis following implant-based breast reconstruction: what is the evidence?. Plast Reconstr Surg.

[12] Phillips B.T., Bishawi M., Dagum A.B., Khan S.U., Bui D.T. (Jan. 2013). A systematic review of antibiotic use and infection in breast reconstruction: what is the evidence?. Plast Reconstr Surg.

[13] Hsieh H.-H., Liu P.-H., Chang C.-J., Kuo Y.-L., Chang T.-Y. (Aug. 2024). Effectiveness of extended antibiotic prophylaxis in implant-based breast reconstruction surgery: A meta-analysis. J Plast Reconstr Aesthet Surg.

[14] Kanapathy M., Faderani R., Arumugam V., Haque S., Mosahebi A. (Nov. 2021). Management of periprosthetic breast infection: a systematic review and meta-analysis. J Plast Reconstr Aesthet Surg.

[15] Bhardwaj S. (Aug. 2022). Antibiotics and antibiotic resistance- flipsides of the same coin. Curr Pharm Des.

[16] Spear S.L., Seruya M. (Apr. 2010). Management of the infected or exposed breast prosthesis: A single surgeonʼs 15-year experience with 69 patients. Plast Reconstr Surg.

[17] Agarwal S., Ettinger R.E., Kung T.A., Kozlow J.H., Brown D.L. (July 2017). Cohort study of immediate implant exchange during acute infection in the setting of breast reconstruction. J Plast Reconstr Aesthet Surg.

[18] Dobke M., Hauch A., Crowley J. (Aug. 2020). Subclinical infection of the silicone breast implant surface as a possible cause of capsular contracture: A follow-up. Aesthet Plast Surg.

[19] Mempin M., Hu H., Chowdhury D., Deva A., Vickery K. (Nov. 2018). The A, B and C’s of silicone breast implants: anaplastic large cell lymphoma, biofilm and capsular contracture. Materials.

[20] Olsen M.A. (Jan. 2008). Hospital-associated costs due to surgical site infection after breast surgery. Arch Surg.

[21] Song D.H., Ooi A.S. (Sept. 2016). Reducing infection risk in implant-based breast-reconstruction surgery: challenges and solutions. Breast Cancer Targets Ther.

[22] Caputo Glenda Giorgia (2025). Redefining infection management in implant-based breast reconstruction: insights and innovations from an 11-year retrospective analysis. J Plast Reconstr Aesthet Surg: JPRAS.

[23] Caputo Glenda Giorgia (29 Dec. 2025). Rapid molecular diagnosis of breast implant infections: A new frontier in plastic surgery. Plast Reconstr Surg.

[24] Monari M., Vaccari S., Lupacchini A.M., Parisi A., Zolesi N., Beltrame S., Petrillo P., Grizzi F., Hegazi MAAA, Giuli R.D., Klinger F., Rusconi R., Vinci V. (2026 Feb). Early detection of viable microorganisms in "sterile" periprosthetic fluids in implant-based breast reconstruction: A bioelectrochemical approach using screen-printed electrodes. J Plast Reconstr Aesthet Surg.

[25] Mano M.S., Çitaku F.T., Barach P. (Jan. 2022). Implementing multidisciplinary tumor boards in oncology: a narrative review. Future Oncol.

[26] Pillay B. (Jan. 2016). The impact of multidisciplinary team meetings on patient assessment, management and outcomes in oncology settings: A systematic review of the literature. Cancer Treat Rev.

